# Control of tissue morphogenesis by the HOX gene *Ultrabithorax*

**DOI:** 10.1242/dev.184564

**Published:** 2020-03-02

**Authors:** Maria-del-Carmen Diaz-de-la-Loza, Ryan Loker, Richard S. Mann, Barry J. Thompson

**Affiliations:** 1 Epithelial Biology Laboratory, The Francis Crick Institute, 1 Midland Rd, St Pancras, London NW1 1AT, United Kingdom; 2 Department of Neuroscience, Mortimer B. Zuckerman Mind Brain Behavior Institute, Columbia University, New York, NY 10027, USA; 3 Department of Biochemistry and Molecular Biophysics, Columbia University, New York, NY 10027, USA; 4 EMBL Australia, The John Curtin School of Medical Research, The Australian National University, Acton, Canberra, ACT 2601, Australia

**Keywords:** *Drosophila*, Development, Morphogenesis, *Stubble*, *Notopleural*

## Abstract

Mutations in the *Ultrabithorax* (*Ubx*) gene cause homeotic transformation of the normally two-winged *Drosophila* into a four-winged mutant fly. *Ubx* encodes a HOX family transcription factor that specifies segment identity, including transformation of the second set of wings into rudimentary halteres. *Ubx* is known to control the expression of many genes that regulate tissue growth and patterning, but how it regulates tissue morphogenesis to reshape the wing into a haltere is still unclear. Here, we show that *Ubx* acts by repressing the expression of two genes in the haltere, *Stubble* and *Notopleural*, both of which encode transmembrane proteases that remodel the apical extracellular matrix to promote wing morphogenesis. In addition, *Ubx* induces expression of the *Tissue inhibitor of metalloproteases* in the haltere, which prevents the basal extracellular matrix remodelling necessary for wing morphogenesis. Our results provide a long-awaited explanation for how Ubx controls morphogenetic transformation.

## Abstract (precis)

**Highlighted Article:** The HOX gene *Ubx* governs the wing-to-haltere transformation in dipteran insects by regulating expression of proteases that control ECM remodelling to disrupt wing elongation and flattening.

## INTRODUCTION

The animal kingdom has evolved an astonishing variety of patterns, sizes and shapes. In insect evolution, it is widely understood that the last common ancestor of all flying insects was four-winged, as evidenced by the fossil record, with modern two-winged *Drosophila* (order Diptera, ‘true flies’) arising later alongside many more abundant four-winged species (Carroll, 1995; Carroll et al., 1995). In place of the second wing pair, Dipterans exhibit a pair of rudimentary stumps known as ‘halteres’, which are thought to function as balancing organs during flight. This evolutionary wing-to-haltere transformation is considered an example of ‘homeosis’ [Greek for ‘replacement’; a term coined by William Bateson in 1894 (Bateson, 1894)] and the discovery of the classic bithorax-complex (BX-C) mutants by Calvin Bridges were the first examples of ‘homeotic’ transformation (Bridges, 1944), characterised in detail by Ed Lewis (Lewis, 1963, 1978, 1998). Within the BX-C, *Ultrabithorax* (*Ubx*) is the key homeotic gene orchestrating wing-to-haltere transformation and encodes a transcription factor containing a highly conserved DNA binding domain named the ‘homeobox’ that is found throughout the HOX family of transcription factors (Affolter et al., 1990a,b, 2008; Akam et al., 1984; Akam, 1983; Beachy et al., 1985; Bender et al., 1983; Casanova et al., 1985; Chan et al., 1994; Chan and Mann, 1993; Desplan et al., 1988; Gehring, 1992; Mann and Hogness, 1990; McGinnis et al., 1984a,b; Sánchez-Herrero et al., 1985; Scott and Weiner, 1984; Struhl, 1982).

In *Drosophila*, mutations in *Ubx* alter the identity of an entire segment of the body plan, namely transformation of the third thoracic segment into a duplicated second thoracic segment (Bridges, 1944; Lewis, 1963, 1978, 1998). Ubx is strongly expressed in the third thoracic segment throughout development, beginning in the embryo upon subdivision of the anterior-posterior (A-P) body axis (Akam, 1983; Beachy et al., 1985), where it influences segmental patterning of cuticular denticle belts (Crocker et al., 2015). There is also some expression of Ubx in the abdominal segments, where it cooperates with two other BX-C transcription factors Abd-A and Abd-B to alter denticle belt pattern and represses appendage formation in the abdomen (Akam and Martinez-Arias, 1985; Beachy et al., 1985; Carroll, 1995; Castelli-Gair et al., 1994; Delorenzi and Bienz, 1990; Gebelein et al., 2002; Panganiban et al., 1997; Vachon et al., 1992; Warren et al., 1994; White and Wilcox, 1985). In the third thoracic segment, the expression of Ubx leads to the dramatic transformation of the second pair of wings into halteres, but has more subtle effects on development of the legs, which are relatively similar between segments except for differences in size and in the pattern of bristles (Casanova et al., 1985; Davis et al., 2007; Lawrence et al., 1979; Rozowski and Akam, 2002; Stern, 1998; Struhl, 1982). Thus, Ubx must induce tissue-specific transcriptional changes that prevent wing formation without affecting leg formation.

As the *Ubx* gene is expressed in similar or overlapping patterns in both *Drosophila* and four-winged insects, such as butterflies, it must be that evolutionary acquisition of new wing-specific Ubx target genes is responsible for the loss of the second pair of wings in dipterans such as *Drosophila* (Carroll, 1995; Warren et al., 1994). Efforts to identify the Ubx target genes responsible for transforming a wing into a haltere have uncovered many genes with important roles in governing wing growth and pattern (Agrawal et al., 2011; Crickmore and Mann, 2006, 2007; Galant et al., 2002; Makhijani et al., 2007; Mohit et al., 2006; Pallavi et al., 2006; Pavlopoulos and Akam, 2011; Prasad et al., 2003; Shashidhara et al., 1999; Weatherbee et al., 1998). In contrast, the identity of Ubx target genes that govern wing morphology is still unclear. Thus, how Ubx induces a morphogenetic change in shape – from an elongated and flattened wing blade to a stumpy haltere – remains a fundamental unsolved problem.

It was recently reported that Ubx may alter wing morphogenesis by repressing expression of a matrix metalloprotease (Mmp1) in the haltere, as determined by immunostaining with an anti-Mmp1 antibody (De Las Heras et al., 2018). However, loss of Mmp1 does not impair wing morphogenesis, owing to compensation by Mmp2, suggesting that other target genes must mediate the function of Ubx in controlling wing morphogenesis. We recently discovered that proteolytic remodelling of both the apical extracellular matrix [aECM; composed of ZP-domain proteins such as Dumpy (Dp)] and the ‘basement membrane’ basal extracellular matrix (bECM; composed of Collagen IV, Laminin and Perlecan) are crucial for wing morphogenesis, and that both remodelling processes are repressed by Ubx in the haltere (Diaz-de-la-Loza et al., 2018). We now demonstrate that Ubx acts by specifically repressing expression of two genes encoding aECM proteases: *Stubble* (*Sb*) and *Notopleural* (*Np*) and by inducing expression of a third gene encoding a bECM protease (Mmp1/2) inhibitor: *Tissue inhibitor of metalloproteases* (*Timp*).

## RESULTS

### Ultrabithorax represses the expression of *Sb* and *Np* to impair apical ECM degradation in the haltere

**Fig. 1.**
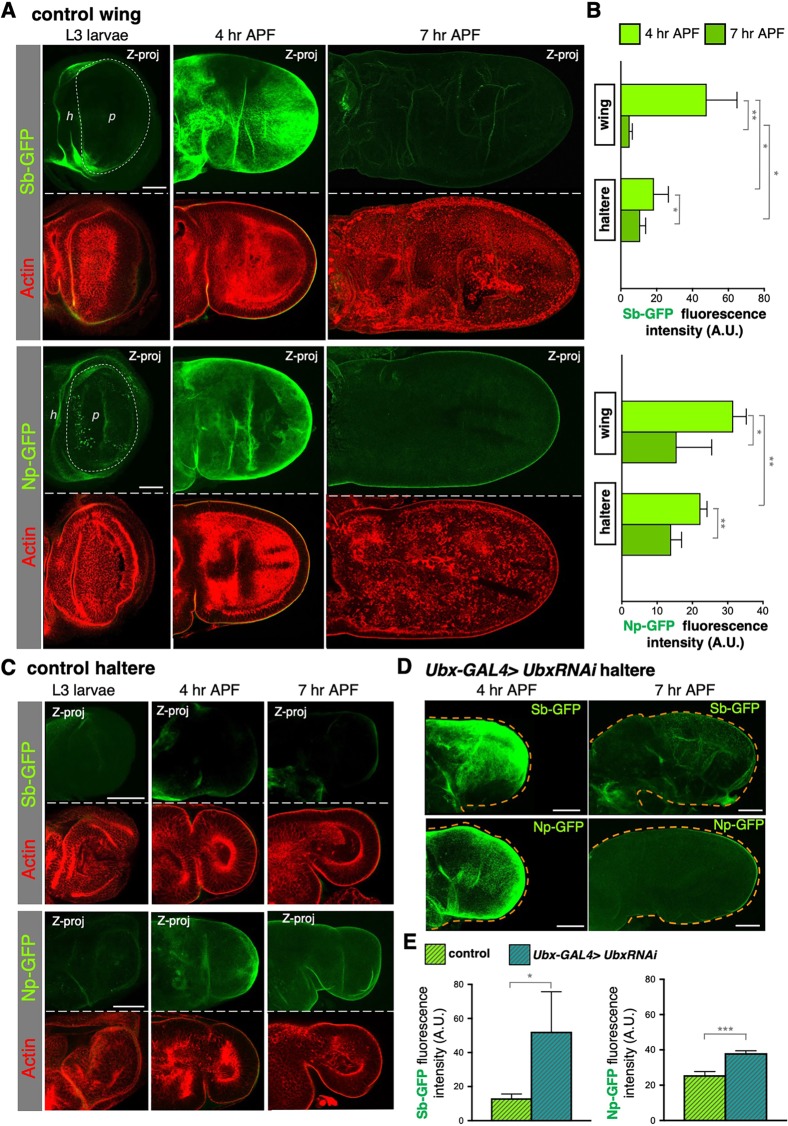
**Ubx is required to repress expression of the Sb and Np aECM proteases in the haltere.** (A) Maximum projection (Z-proj) of endogenous Stubble-GFP (Sb-GFP; top) and Notopleural-GFP (Np-GFP; bottom) localisation in developing wings at third instar larvae (L3), 4 h and 7 h APF. Wing hinge (h) and pouch (p) regions in L3 wing discs are indicated. Sb and Np start to be expressed at the end of the larval stage, mostly visible at the hinge folds. At 4 h APF, before ECM degradation, they strongly localise at the apical membrane of the wing epithelium, and their levels decrease in 7 h APF wings, which have already expanded and elongated after the ECM is degraded. Actin cytoskeleton is shown in red. (B) Quantification of Sb-GFP and Np-GFP immunofluorescence signal in the wing and the haltere at 4 h and 7 h APF. Both proteins are strongly expressed in the wing compared with the haltere. Wings show a maximum of GFP fluorescence at 4 h APF, whereas haltere fluorescence levels remain low. (C) Maximum projection (Z-proj) of Sb-GFP and Np-GFP localisation in developing halteres from third instar larvae (L3), at 4 h and 7 h APF. Halteres show consistently low levels of Np-GFP and Sb-GFP during all developmental stages. Actin cytoskeleton is shown in red. (D) Maximum projections of Sb-GFP (top) and Np-GFP (bottom) in *ubxRNAi*-expressing halteres (*ubx-Gal4>UAS.UbxRNAi*) at 4 h and 7 h APF. Loss of Ubx restores high levels of Sb and Np in the haltere at 4 h APF, leading to ectopic ECM degradation, which results in flattened and expanded halteres at 7 h APF. Dashed lines indicate the perimeter of the haltere, determined by looking at the actin cytoskeleton. (E) Quantification of Sb-GFP and Np-GFP immunofluorescence signal in control and *ubx-Gal4>UAS.UbxRNAi* wings and halteres at 4 h APF. Depletion of Ubx increases Sb-GFP and Np-GFP expression in the haltere to similar levels to the wing. Data are mean±s.d., *n*>4 for each developmental stage. *P<0.05, **P<0.005, ***P<0.001 (two-tailed Student's *t*-test). Scale bars: 50 μm.

We began by using CRISPR to generate endogenously tagged Green Fluorescent Protein (GFP) knock-in fusion protein alleles: *Sb-GFP* and *Np-GFP*. We found that both Sb-GFP and Np-GFP start to be expressed in the developing wing primordium (known as an ‘imaginal disc’) at the end of the third larval instar (L3), immediately before the initiation of aECM degradation and consequent pupal wing morphogenesis (Fig. 1A,B). At late L3 wing Sb-GFP and Np-GFP are detectable in the hinge folds which surround the wing pouch, the wing disc region that will give rise to the adult wing. The expression of these two aECM proteases reaches its maximum at 4 h after puparium formation (APF) when early metamorphosis ECM degradation occurs, and it decreases again from 4-7 h APF. Importantly, both Sb-GFP and Np-GFP are partially repressed in the haltere (Fig. 1B,C) and this repression requires Ubx (Fig. 1D,E). Notably, Sb-GFP and Np-GFP are not repressed in the leg imaginal discs at this stage, consistent with the notion that Ubx acts to alter morphogenesis in the haltere by modifying target gene expression specifically in this tissue type (Fig. S1).

**Fig. 2.**
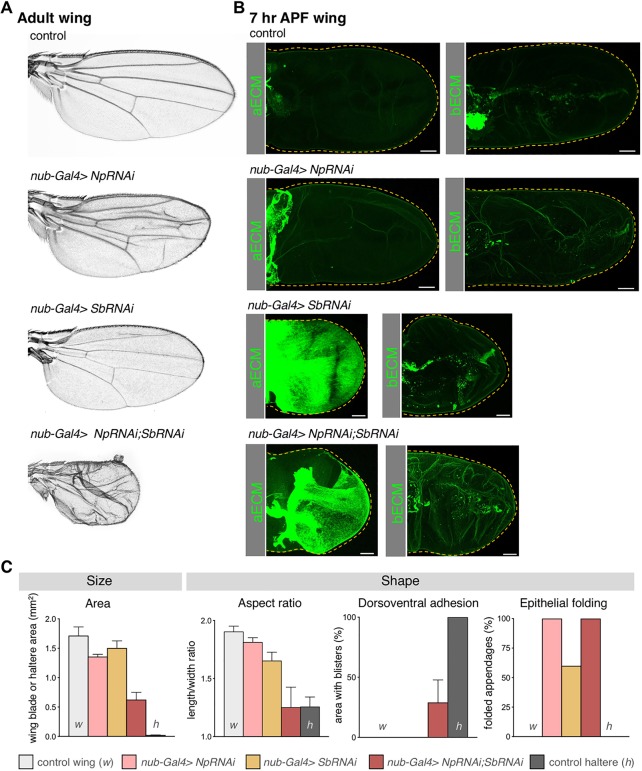
**Depletion of Sb and Np impairs** **aECM** **remodelling and wing morphogenesis.** (A) Adult wings from control animals and from animals with depletion of Np (*nub-Gal4>NpRNAi*), Sb (*nub-Gal4>SbRNAi*) or both (*nub-Gal4>NpRNAi;SbRNAi*). Simultaneous depletion of Sb and Np apical proteases during metamorphosis results in smaller and rounded wings. (B) Maximum projections of 7 h APF wings in control and mutant conditions with low levels of Np and Sb expressing Dp-YFP (aECM) or Vkg-GFP (bECM). Np depletion does not affect aECM degradation, and wings have elongated and expanded normally at 7 h APF; however, depleting Sb strongly impairs aECM degradation and wing expansion at 7 h APF, consistent with the strong expression of Sb at this stage of development. As loss of both Sb and Np is required to affect the adult wing, Np must function after 7 h APF to degrade the aECM, even in the absence of Sb (see Figs S1 and S2). Note that bECM degradation is not affected by depletion of apical proteases. Dashed lines indicate the perimeter of the wing blade, determined by looking at the actin cytoskeleton. (C) Quantification of size (area) and shape characteristics (aspect ratio, dorsoventral adhesion and epithelial folding) in control (w), *nub-Gal4>NpRNAi*, *nub-Gal4>SbRNAi* and *nub-Gal4>NpRNAi;SbRNAi* wings compared with control halteres (h). Mean±s.d. are shown from up to 20 wings or halteres for each genotype. Inhibition of aECM degradation by depletion of apical proteases decreases wing area and elongation, and impairs the adhesion of the dorsoventral layers, all features present in the haltere. aECM depletion also results in folding of the wing blade. Scale bars: 50 μm.

**Fig. 3.**
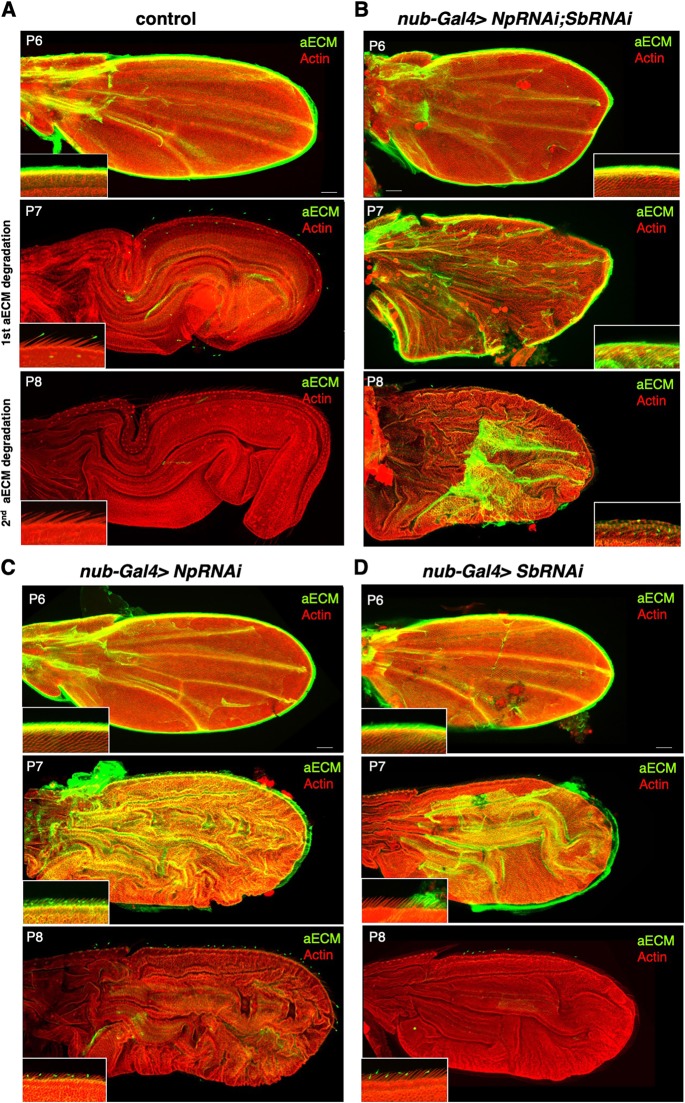
**Apical ECM is degraded at late metamorphosis by Sb and Np.** (A) Dumpy-YFP (Dp-YFP)-labelled aECM is normally degraded during pupal days P7 and P8. Actin is shown in red. (B) Silencing of both Sb and Np expression by RNAi with *nub-Gal4* prevents aECM degradation at P7 and P8. (C) Silencing of Np expression alone by RNAi with *nub-Gal4* prevents aECM degradation at P7 but not P8. (D) Silencing of Sb expression alone by RNAi with *nub-Gal4* prevents aECM degradation at P7 but not P8. Insets show high magnification views of the pupal wing margin. Note the proximity of the wing margin bristles to the aECM in the control. Scale bars: 50 µm.

We next sought to test whether *Sb* and *Np* are important for wing morphogenesis, and specifically whether repression of *Sb* and *Np* would be sufficient to explain how Ubx prevents the normal morphogenetic elongation of the wing during development. We began by silencing *Sb* expression using RNA interference (RNAi) in a tissue-specific fashion using a wing-specific *nubbin.Gal4* (*nub.Gal4*) driver transgene in combination with GAL4-dependent *UAS.SbRNAi* inverted-repeat hairpin RNAi inducing transgene. Silencing of *Sb* alone strongly increases the levels of aECM surrounding the wing during the early hours of pupal wing morphogenesis (4-7 h APF) (Diaz-de-la-Loza et al., 2018), but ultimately gives rise to a normally shaped wing (Fig. 2). This finding suggests that another aECM protease may compensate for the loss of *Sb*, and primarily acts later in development to degrade the aECM and allow wing elongation. Accordingly, during late pupal development (P7 to P8 pupal stages, 40-48 h APF), a second aECM remodelling event occurs to allow further wing expansion (Figs S2 and S3). P6 pupal wings are surrounded by a new layer of Dumpy secreted from 8 h APF, which links the apical side of the wing epithelia with the encapsulating cuticle (Ray et al., 2015). We found that from P6 to P7 the totality of the apical ECM that surrounds the wing is degraded in two consecutive steps. Such degradation is essential to allow wing expansion, as inhibition of Dumpy degradation by silencing of *Sb* and/or *Np* impairs wing expansion inside the cuticle, leading to smaller, rounder and folded P7 wings (Fig. 3). As expected, we found that the late round of aECM degradation is also necessary to allow the elongation of bristles at the wing margin and the thorax (explaining the classic *Sb* haploinsufficiency phenotype) (Fig. S3). Np appears to be the key missing protease, as silencing of both *Sb* and *Np* by RNAi prevents formation of a normal adult wing, instead generating a reduced structure that has failed to fully expand or elongate during either early or late stages of metamorphosis (Figs 2 and 3). Indeed, it was recently shown that *Np* is necessary to degrade Dumpy in the *Drosophila* embryo (Drees et al., 2019).

**Fig. 4.**
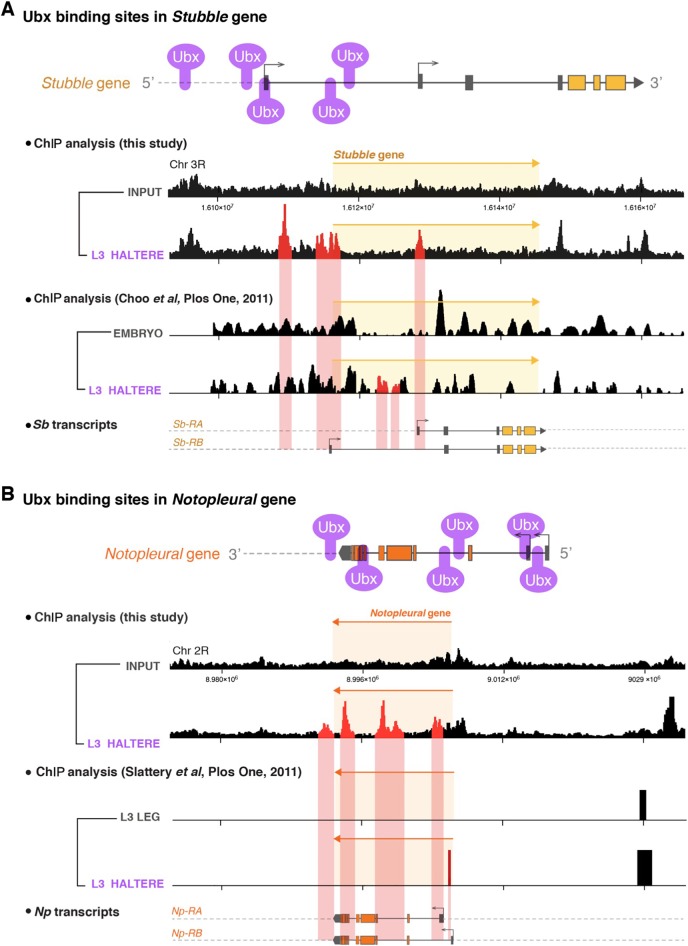
**Ubx binds to specific sites in the *Sb* and *Np* genes in the haltere.** (A) Ubx binding sites in the *Sb* gene from ChIP experiments performed in this study and data mined from the previous ChIP dataset published in Choo et al. (2011). To identify specific Ubx DNA binding sites in third instar larvae (L3) halteres, we extracted chromatin from L3 halteres and compared Ubx binding peaks in samples with and without adding the antibody to pulldown Ubx. In the previous dataset (Choo et al., 2011), Ubx binding peaks in L3 halteres were compared with whole-embryo extracts. We found four haltere-specific Ubx binding peaks in *Sb* (highlighted in red) located at 5′ intergenic regions and introns. (B) Ubx binding sites in the *Np* gene from ChIP experiments performed in this study and data mined from the previous ChIP dataset published in Choo et al. (2011). To look for specific Ubx DNA binding sites in third instar larvae (L3) halteres, we extracted chromatin from L3 halteres and compared Ubx binding peaks in samples with and without adding the antibody to pulldown Ubx. In the previous dataset, Ubx binding peaks in L3 halteres were compared with L3 leg samples. We found five haltere-specific Ubx binding peaks at *Np* (highlighted in red) located at 5′ and 3′ intergenic regions. Annotation of genomic location and protein isoforms were adapted from the Flybase database (https://flybase.org/).

Next, we sought to investigate whether Ubx was directly controlling *Sb* and *Np* expression via genome-wide chromatin immunoprecipitation (ChIP) experiments in which we analysed specific Ubx DNA binding sites in chromatin extracted from in L3 halteres (Fig. 4). We found that Ubx binds directly to the *Sb* and *Np* regulatory regions, confirming and extending what was found in two previous genome-wide ChIP studies (Choo et al., 2011; Slattery et al., 2011). Together, the above results show that repression of both *Sb* and *Np* is sufficient to disrupt morphogenesis of the adult wing, confirming their importance as downstream effector genes of Ubx.

### Ubx impairs basal ECM degradation in the haltere by activating the expression of *Timp* and repressing the expression of *Mmp1* and *Mmp2*

**Fig. 5.**
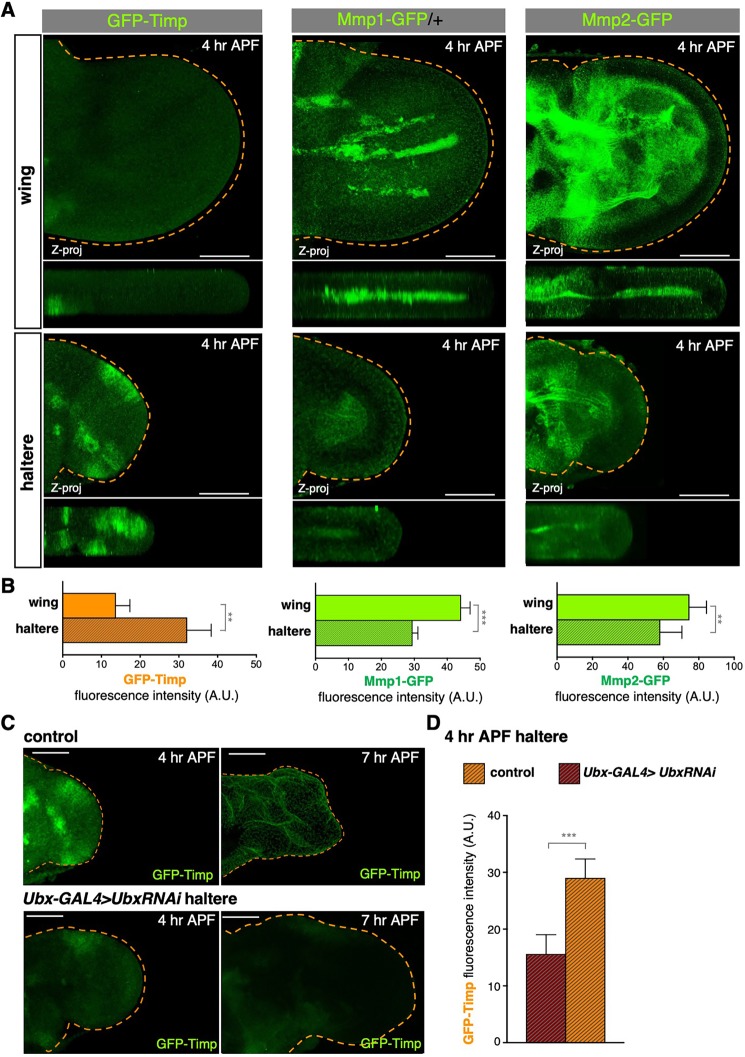
**Ubx is required for expression of Timp in the haltere.** (A) Maximum projection (Z-proj; top) and cross-sections (bottom) of 4 h wings and halteres expressing endogenous *GFP-Timp*, *Mmp1-GFP* or *Mmp2-GFP*. GFP-Timp is not detectable in the wing blade but is strongly expressed in specific regions of the haltere. Mmp2-GFP and Mmp1-GFP accumulate at the basal membrane of both the wing and the haltere. (B) Quantification of GFP-Timp, Mmp-GFP and Mmp2-GFP immunofluorescence signal in wings and halteres at 4 h APF. Halteres show higher levels of GFP-Timp and lower levels of Mmp1-GFP and Mmp2-GFP compared with the wing. (C) Maximum projections of GFP-Timp of control (*ubx-Gal4/+*) and *ubxRNAi*-expressing halteres (*ubx-Gal4>UAS.UbxRNAi*) at 4 h and 7 h APF. Depletion of Ubx in the haltere decreases GFP-Timp expression at 4 h APF, immediately before ectopic ECM degradation in mutant halteres. (D) Quantification of GFP-Timp immunofluorescence signal in control and *ubx-Gal4>UAS.UbxRNAi* wings and halteres at 4 h APF. Depletion of Ubx decreases GFP-Timp expression compared with control halteres. Data are mean±s.d., *n*>4 for each developmental stage. **P<0.005, ***P<0.001 (two-tailed Student's *t*-test). Dashed lines indicate the perimeter of the wing blade, determined by looking at the actin cytoskeleton. Scale bars: 50 μm.

**Fig. 6.**
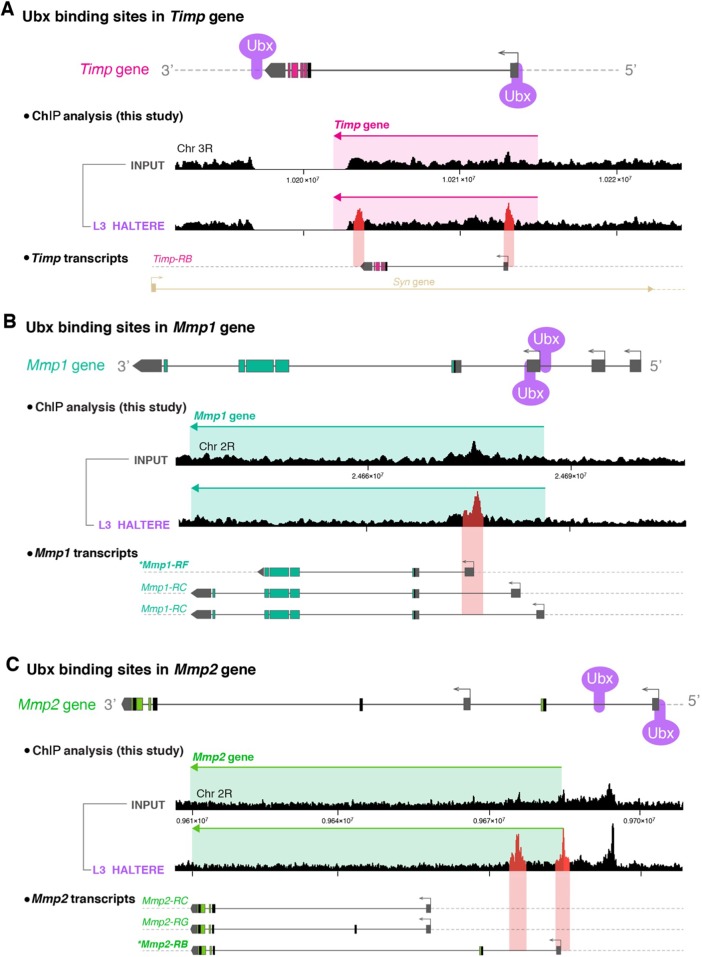
**Ubx binds to specific sites in the *Timp*, *Mmp1* and *Mmp2* genes in the haltere.** (A) Ubx binding sites in the *Timp* gene from ChIP experiments performed in this study. To look for specific Ubx DNA binding sites in third instar larvae (L3) halteres, we extracted chromatin from L3 halteres and compared Ubx binding peaks in samples with and without adding the antibody to pulldown Ubx. We found two haltere-specific Ubx binding peaks for *Timp* (highlighted in red), located at 5′ and 3′ regulatory regions. (B) Ubx binding sites in the *Mmp1* gene from ChIP experiments performed in this study. We found two haltere-specific Ubx binding peaks for *Mmp1* (highlighted in red), located at 5′ intergenic regions or introns. The *Mmp1-RF* isoform that carries the GFP insertion in our *Mmp1-GFP* knock-in is marked with an asterisk. (C) Ubx binding sites in the *Mmp2* gene from ChIP experiments performed in this study. We found two haltere-specific Ubx binding peaks for *Mmp2* (highlighted in red), located at 5′ intergenic regions or introns. The *Mmp2-RB* isoform carrying the GFP insertion in our *Mmp2-GFP* knock-in is marked with an asterisk. Annotation of genomic location and protein isoforms were adapted from Flybase database (https://flybase.org/).

As mentioned above, Ubx also prevents the haltere from remodelling the bECM, which is composed of Collagen IV [α2 subunit encoded by the *viking* (*vkg*) gene and α1 subunit encoded by *Col4A1* (also known as *Cg25*)], Laminin and Perlecan. It is therefore tempting to speculate that Ubx might directly repress expression of the bECM protease genes *Mmp1* or *Mmp2*, as recently reported for *Mmp1* (De Las Heras et al., 2018). However, it is also possible that Ubx acts indirectly to inhibit Mmp1/2 activity by inducing expression of their inhibitor *Timp*. To distinguish between these possibilities, we used CRISPR to generate three endogenously GFP-tagged fusion protein alleles: *GFP-Timp*, *Mmp1-GFP* and *Mmp2-GFP*. We find that the Mmp1-GFP and Mmp2-GFP proteins are expressed in both the wing and the haltere, although with lower levels in the haltere, particularly for Mmp1-GFP (De Las Heras et al., 2018), whereas GFP-Timp is only expressed in the haltere and not in the developing wing blade (Fig. 5A,B). Silencing of Ubx causes a corresponding loss of GFP-Timp expression in the haltere (Fig. 5C,D). Consistent with repression of *Mmp1* and *Mmp2* as well as activation of *Timp* in the haltere, we found that Ubx also binds directly to those genes in L3 halteres as shown in our ChIP data (Fig. 6). These results show that Ubx acts not only via direct repression of *Mmp1/2* but also indirectly via upregulation of *Timp* expression to inhibit Mmp1/2-mediated bECM matrix remodelling in the haltere. Although Ubx is generally a transcriptional repressor, there are precedents for Ubx acting as an activator for certain target genes, and *Timp* may be one such example (Zandvakili et al., 2019). Importantly, Ubx-dependent induction of *Timp* is specific to the haltere and does not occur in the leg epithelium, despite expression of GFP-Timp in the tendon (Fig. S1C).

### Prevention of ECM degradation in the wing impairs wing morphogenesis

**Fig. 7.**
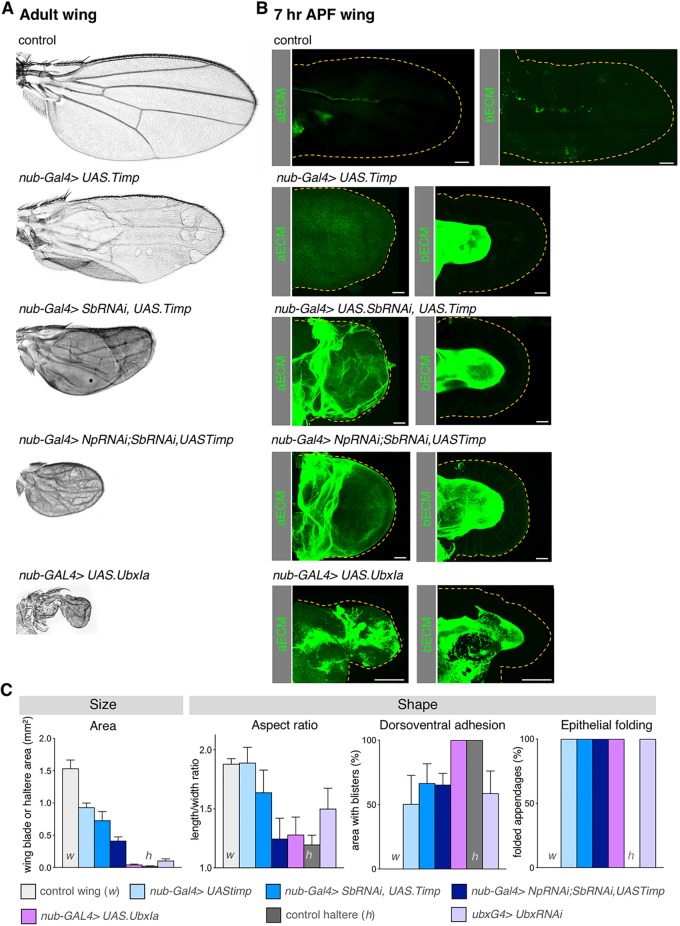
**Preventing both basal and apical ECM remodelling strongly impairs wing morphogenesis.** (A) Adult wings from control and animals overexpressing Timp (*nub-Gal4>UAS.Timp*), combined with the depletion of Sb (*nub-Gal4>SbRNAi,UAS.Timp*) or both Sb and Np (*nub-Gal4>Np.RNAi;SbRNAi,UAS.Timp*), compared with wings ectopically expressing a *Ubx* allele (*nub-Gal4>UbxI^a^*). Reduction in the activity or expression of both aECM and bECM proteases dramatically decreases wing size and length, resembling the wing-to-haltere transformation phenotype caused by UbxI^a^ overexpression. (B) Maximum projections of 7 h APF wings from control and animals overexpressing Timp, combined with the depletion of Sb and Np, compared with wings ectopically expressing the *UbxI^a^* allele. Overexpression of Timp inhibits bECM degradation, whereas overexpression of Sb inhibits aECM degradation, impairing wing expansion and elongation (see Fig 1B), similar to UbxI^a^-expressing 7 h APF mutant wings. (C) Quantification of size (area) and shape characteristics (aspect ratio, dorsoventral adhesion and epithelial folding) in control (w), *nub-Gal4>UAS.Timp*, *nub-Gal4>SbRNAi,UAS.Timp*, *nub-Gal4>Np.RNAi;SbRNAi,UAS.Timp* and *nub-Gal4>UbxI^a^* wings compared with control (h) and *Ubx-Gal4>Ubx.RNAi* halteres. Inhibition of bECM degradation by *Timp* overexpression reduces wing size and dorsoventral adhesion. Data are mean±s.d. from up to 20 wings or halteres for each genotype. When combined with depletion of apical proteases, the defects associated with impaired bECM degradation in the wing increase and includes wing rounding, similar to wings ectopically expressing *Ubx* in the wing and control halteres. bECM depletion also results in folding of the wing blade. Scale bars: 50 μm.

We next sought to test whether repression of *Sb* and *Np* expression combined with inhibition of Mmp1/2 activity via induction of *Timp* expression would help explain how Ubx prevents wing morphogenesis. The *nub.Gal4* driver was used to promote wing-specific expression of *UAS.Sb-IR* and *UAS.Np-IR* hairpin RNAi transgenes in various combinations with a *UAS.Timp* inducible transgene (Fig. 7). Accordingly, we found that silencing of both *Sb* and *Np* by RNAi combined with Timp overexpression is sufficient to strongly disrupt wing morphogenesis, producing a small stump: wings three times smaller than controls, rounder (with a similar aspect ratio to control haltere), with ∼70% of the wing area inflated – all features of adult halteres (Fig. 7), and very similar to the phenotype caused by Ubx overexpression specifically during the pupal stages of development (Roch and Akam, 2000). Inhibiting ECM degradation most likely reduces wing size by affecting wing expansion and elongation during both early and late metamorphosis, which also causes wing folding, and by impairing the adhesion of dorsal and ventral wing layers, leading to the presence of blisters (Fig. 7A,C). As expected, the reduction in area is not as strong as in wings overexpressing the *UbxI^a^* allele or control halteres, as Ubx also reduces cell proliferation (Agrawal et al., 2011; Makhijani et al., 2007; Mohit et al., 2006; Pallavi et al., 2006; Pavlopoulos and Akam, 2011; Prasad et al., 2003; Shashidhara et al., 1999; Weatherbee et al., 1998). These results indicate that *Sb*, *Np* and *Timp* are the key genes regulated by Ubx that prevent matrix remodelling and disrupt morphogenetic elongation and flattening during the wing-to-haltere transformation.

We next performed a further test of the role of *Sb*, *Np* and *Timp* as downstream effectors of Ubx. This is possible because Ubx is normally expressed in the peripodial epithelium that surrounds the developing wing disc during the larval stages. The peripodial epithelium of the wing is dramatically removed in one collective motion at the onset of matrix remodelling and wing morphogenesis at the beginning of pupal development (4 h APF). Importantly, the matrix remodelling observed in the wing disc proper at this stage does not occur in peripodial epithelium, which remains covered in matrix even as it is removed from the disc proper (Fig. S4, Movies 1 and 2). The expression of Ubx in the peripodial epithelium correlates with the absence of Sb-GFP and Np-GFP as well as expression of GFP-Timp (Fig. S4). Thus, the peripodial epithelium and haltere both exhibit a similar program of Ubx-regulated *Sb*, *Np* and *Timp* expression, which explains why neither the apical nor basal matrix is remodelled in these tissues at this stage of development.

Finally, we sought to examine whether the principles we have uncovered might be conserved in mammals. Remodelling of the basement membrane bECM components (collagen IV, laminin, perlecan) by MMPs and TIMPs has been well studied in mammals and found to be crucial for morphogenesis (Hynes, 2009). However, the components of the aECM (ZP-domain proteins) and their corresponding Sb-family proteases are also highly conserved across the animal kingdom (Fig. S5) (Plaza et al., 2010). Whether patterned expression of the aECM proteases might be responsible for the pattern of aECM distribution in mammalian tissues is completely unexplored. We therefore compared the pattern of the aECM protein UMOD with the Sb protease homologue TMPRSS15 in the human intestine, and found that they exhibit opposing distributions along the crypt-villus axis, such that the aECM ensheaths the villus but is absent at the tips, where cells must be sloughed off to maintain homeostasis (Fig. S5). We found a similar opposing distribution of collagen IV with Mmp15 in the intestinal villus (Fig. S5). These findings suggest that, as in *Drosophila*, morphogenesis of mammalian tissues may also involve remodelling of both aECM and bECM via patterned expression and activity of their respective proteases.

## DISCUSSION

**Fig. 8.**
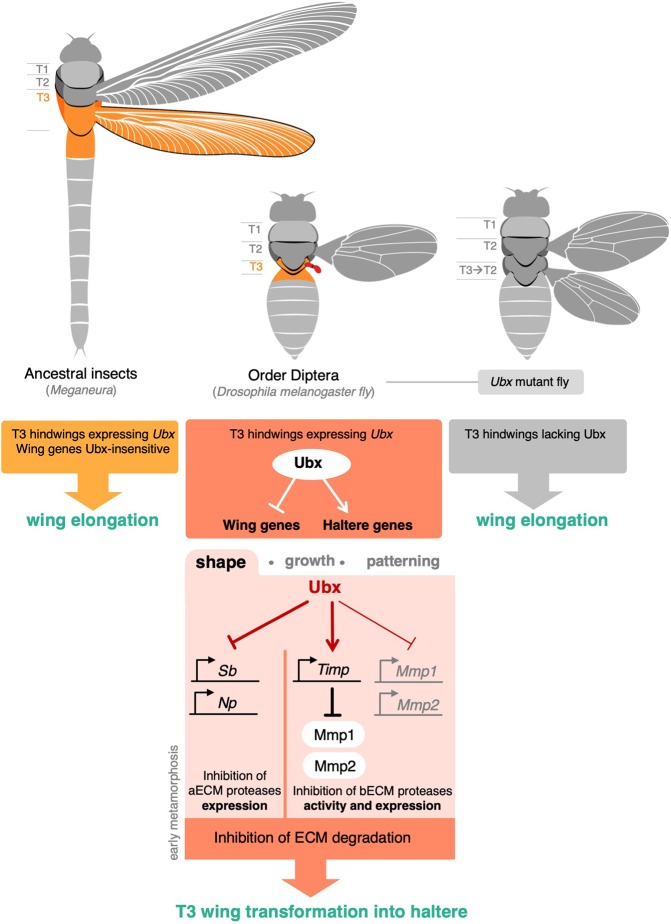
**Ubx controls apical and basal** **ECM degradation to regulate morphogenesis.** Schematic of Ubx expression and function in *Drosophila* and a hypothetical four-winged ancestor. Ubx controls organ shape via regulation of aECM and bECM proteases, in addition to its known functions in regulating organ growth and patterning. These target genes have presumably evolved to be specifically regulated in the *Drosophila* wing and/or haltere, and must be insensitive to Ubx in four-winged ancestors.

Our results reveal how *Ubx* – a homeotic gene that encodes the founding member of the HOX-family of transcription factors – regulates apical and basal matrix remodelling to control epithelial morphogenesis (summarised in Fig. 8). Ubx strongly represses two genes encoding apical matrix proteases (*Np* and *Sb*), as well as partially repressing two genes encoding basal matrix metalloproteases (*Mmp1* and *Mmp2*), while inducing an inhibitor of Mmp1/2 (*Timp*) in the haltere. In this way, Ubx prevents both apical and basal matrix remodelling in the haltere, a key event in the homeotic wing-to-haltere transformation. In addition to regulating morphogenesis, Ubx controls many other genes affecting wing growth and pattern (Agrawal et al., 2011; Crickmore and Mann, 2006, 2007; Galant et al., 2002; Makhijani et al., 2007; Mohit et al., 2006; Pallavi et al., 2006; Pavlopoulos and Akam, 2011; Prasad et al., 2003; Shashidhara et al., 1999; Weatherbee et al., 1998). Together, the combined repression of morphogenesis, growth and patterning by Ubx is responsible for the full transformation of wing to haltere.

Our findings also support the general view that transcriptional control of matrix synthesis and degradation is a conserved mechanism by which information encoded in the genome is deployed to govern the shape of tissues and organs in animals. Although this concept is broadly appreciated for the regulation of the bECM, the notion that the aECM is also developmentally regulated during tissue morphogenesis needs further investigation, particularly in mammals (Fig. S5). Beyond animals, morphogenesis of plants, fungi and bacteria is also known to be fundamentally dependent on patterned synthesis and degradation of the cell wall, a type of ECM. Thus, genetic control of the matrix appears to be a general principle that shapes all life forms.

## MATERIALS AND METHODS

### *Drosophila* genomic engineering

To analyse *Sb*, *Np*, *Timp*, *Mmp1* and *Mmp2* expression and localisation, five new fly lines were generated using CRISPR-Cas9 directed homologous recombination, inserting GFP in the genome in frame with the endogenous open reading frame (ORF) of each protein. For *Sb*, *Np* and *Timp*, flies with GFP insertions at the N terminus (Nt) or at the C terminus (Ct) were tested, and the GFP-positive versions were selected: *Sb-GFP* (Ct-end GFP insertion), *Np-GFP* (Ct-end GFP insertion) and *GFP-Timp* (Nt-end GFP insertion). For *Mmp1* and *Mmp2*, different GFP insertions were designed based on LaFever et al. (2017), aimed at detecting specific isoforms. We obtained GFP-positive fly lines for *Mmp1-GFP* by tagging its predicted secreted isoform (GFP inserted into the Ct of isoform-RF), and for *Mmp2-GFP* by tagging membrane-tethered Mmp2, which includes a GPI site (isoform-RB, Ct GFP insertion after serine 710).To ectopically express *Sb*, a DNA fragment containing the full length of *Sb* ORF was synthesised (Creative Biogene) and cloned into the *pUASg.attB* vector generating the integration plasmid *pUASg.Sb.attB*. *pUASg.Sb.attB* was then injected into fly embryos to integrate the *UAS.Sb* construct into the genomic DNA at the *attP2* site in the 3rd chromosome via an *attP*/Integrase-mediated reaction.

### *Drosophila melanogaster* genetics

Flies were grown at 25°C using standard procedures. The following fluorescent-tagged proteins were used: Sb-GFP, Np-GFP, GFP-Timp, Mmp1-GFP and Mmp2-GFP (created in this study, see above); Dumpy-YFP (Department of *Drosophila* Genomics and Genetic Resources, 115238) and Collagen IV-GFP (α2 subunit, Vkg-GFP; FlyTrap, G205). Gene expression mediated by the *UAS/Gal4* system was performed at 25°C, using the *ubx-Gal4* driver to direct expression in the haltere, *nub-GAL4* to mediate expression in the wing and *MS1096-Gal4* [Bloomington *Drosophila* Stock Center (BDSC), 8860] in the thorax. *UAS.UbxRNAi* [Vienna *Drosophila* Resource Center (VDRC), 37823], *UAS.SbRNAi* (VDRC, 1613) and *UAS.NpRNAi* (VDRC, 105297) were used to decrease *Ubx*, *Sb* and *Np* expression levels, respectively. Overexpression of Sb and Timp was achieved by *UAS.Sb* (this study) and *UAS.Timp* (BDSC, 58708), respectively.

### Adult tissue preparations

Halteres, wings and legs were dissected from the adult fly, fixed in 70% ethanol and mounted in Hoyer's mounting media, and images were acquired on a Zeiss axioplan microscope with 2.5×/0.075 NA or 10×/0.3 NA objectives, using a LeicaDFC420c digital camera. Thorax images were acquired from flies fixed and immersed in 70% ethanol using a Zeiss Stereo Discovery V20 stereoscope controlled by the Zeiss Zen software using an Axiocam 503 monochrome Zeiss camera. Images were processed using Adobe Photoshop software.

### Immunohistochemistry

White pupae were collected and aged, and then imaginal discs were dissected from the puparium in PBS and transferred to 4% paraformaldehyde for fixation and immunostaining, as previously described (Ray et al., 2015). Anti-GFP antibody (Abcam, ab6662, 1:400) was used to amplify Sb-GFP, Np-GFP, GFP-Timp, Mmp1-GFP, Mmp2-GFP, Dp-YFP and Vkg-GFP fluorescence signals. Mouse anti-Ubx was used at 1:10 (Developmental Studies Hybridoma Bank, FP3.38). The secondary antibody, goat Alexa 546 (Invitrogen, A-11030), was used at 1:500; DAPI (Sigma-Aldrich, D9542) and rhodamine phalloidin 647 (Sigma-Aldrich, 65906) were used at 1:250. Samples were mounted in Vectashield (Vector Labs, H1000) using different separators depending on the thickness of the sample.

### *Ex vivo* culture of pupal imaginal wing discs

Pupal wing discs of the appropriate age were cultured in supplemented Shield and Sang M3 media (Sigma-Aldrich, S8398-1L) as previously described (Diaz-de-la-Loza et al., 2018).

### Live-imaging and imaging of fixed samples

*In vivo* and *ex vivo* samples images were acquired using a Leica SP5 confocal microscope using the 20×/0.70 NA immersion objective, controlled by the Leica Las AF software. Live imaging experiments were performed at room temperature and an average of 50 *z*-sections at 1-2 μm intervals were acquired every 5 min. Images were analysed and processed using Fiji and Adobe Photoshop software.

### Chromatin immunoprecipitation

ChIP-seq experiments were performed as previously described with minor modifications (Oh et al., 2013). Approximately 200 larvae of *yw* genotype were inverted and fixed in crosslinking buffer [10 mM HEPES (pH 8.0), 100 mM NaCl, 1 mM EDTA (pH 8.0), 0.5 mM EGTA (pH 8.0)] containing 1.8% PFA for 20 min at room temperature. Fixed carcasses were then washed twice with buffer-A [10 mM HEPES (pH 8.0), 10 mM EDTA (pH 8.0), 0.5 mM EGTA (pH 8.0), 0.25% Triton X-100] and twice with buffer-B [10 mM HEPES (pH 8.0), 200 mM NaCl, 1 mM EDTA (pH 8.0), 0.5 mM EGTA (pH 8.0), 0.01% Triton X-100]. Haltere discs were removed and placed in sonication buffer [10 mM HEPES (pH 8.0), 1 mM EDTA (pH 8.0), 0.5 mM EGTA (pH 8.0), 1% Triton X-100] containing 0.1% SDS and chromatin was sheared using a Covaris S220 with the setting 105W/2% for 15 min. Samples were pre-cleared using protein-A dynabeads and 5% of each sample was retained for input. Immunoprecipitation was performed using a 1:100 dilution of Ubx antibody (Marin et al., 2012). Protein-A dynabeads were used to purify antibody-bound chromatin and samples were washed and de-crosslinked in parallel with input. Libraries were prepared from purified DNA using New England Biolabs Ultra II Library prep kit and sequenced using an Illumina NextSeq instrument. Libraries were aligned to the dm3 genome using Bowtie2 (Langmead and Salzberg, 2012). Genome browser files were generated using deepTools2 package (Ramírez et al., 2016).

### Human tissue samples

Images of human intestine samples were obtained by datamining the Human Protein Atlas Dataset (www.proteinatlas.org; Uhlén et al., 2005) as follows: UMOD (www.proteinatlas.org/ENSG00000169344-UMOD/tissue/duodenum), TMPRSS15 (www.proteinatlas.org/ENSG00000154646-TMPRSS15/tissue/duodenum), hepsin (www.proteinatlas.org/ENSG00000105707-HPN/tissue/small+intestine), collagen IV (α2 chain, www.proteinatlas.org/ENSG00000134871-COL4A2/tissue/duodenum) and MMP15 (www.proteinatlas.org/ENSG00000102996-MMP15/tissue/duodenum).

### Quantification and statistical analysis

#### Quantification of size and shape of adult wings and halteres

Up to 20 adult wings or halteres were imaged using the Zeiss axioplan microscope with a 2.5×/0.5 NA objective controlled by the Leica Las AF software. Area and maximal width and length were obtained using the ROI tool from Fiji. Aspect ratio was calculated as maximal length/maximal width.

#### Quantification of bristle length at the wing margin

Up to eight adult wings were analysed to measure 100-150 bristles for each genotype. Wing margin regions were imaged using the Zeiss axioplan microscope with a 10×/0.075 NA objective controlled by the Leica Las AF software. For each bristle, maximal length was calculated manually using the ROI tool from Fiji, measuring the length of the shaft from the point at which it emerges from the pocket to its distal apex.

#### Quantification GFP-fusion protein immunofluorescence levels *in vivo*

To examine Sb-GFP, Np-GFP, GFP-Timp, Mmp1-GFP and Mmp2-GFP in the different tissues and experimental conditions, up to eight imaginal discs were analysed per genotype. For every experiment, to be able to compare the fluorescence signal of each GFP-tagged protein between different appendages or experimental conditions, samples were dissected in parallel, fixed and immunostained in the same tube, and images were captured with identical confocal settings, using the distinct morphology of each tissue to distinguish them. For each biological sample, several confocal *z*-stacks acquired at 1.72 µm intervals were selected and projected (maximum intensity projection) to include the complete epithelium. Actin cytoskeleton dye (rhodamine phalloidin) allowed us to detect wing area, and the mean fluorescence intensity in the wing, haltere or leg epithelia was calculated manually using the ROI measurement tool in Fiji.

#### Statistical analysis

Experiments were performed with at least three biological replicates. Mean±s.d. are represented in all graphs. *P<0.05, **P<0.005, ***P<0.0005; two-tailed Student's *t*-tests.

## Supplementary Material

Supplementary information

Reviewer comments
