# Supplementary material for: Control of tissue morphogenesis by the HOX gene Ultrabithorax
Source: Development. 2020 Mar 2;147(5):dev184564. doi: 10.1242/dev.184564 (PMC7063672; doi:10.1242/dev.184564)
Supplement: Supplementary information [file develop-147-184564-s1.pdf]

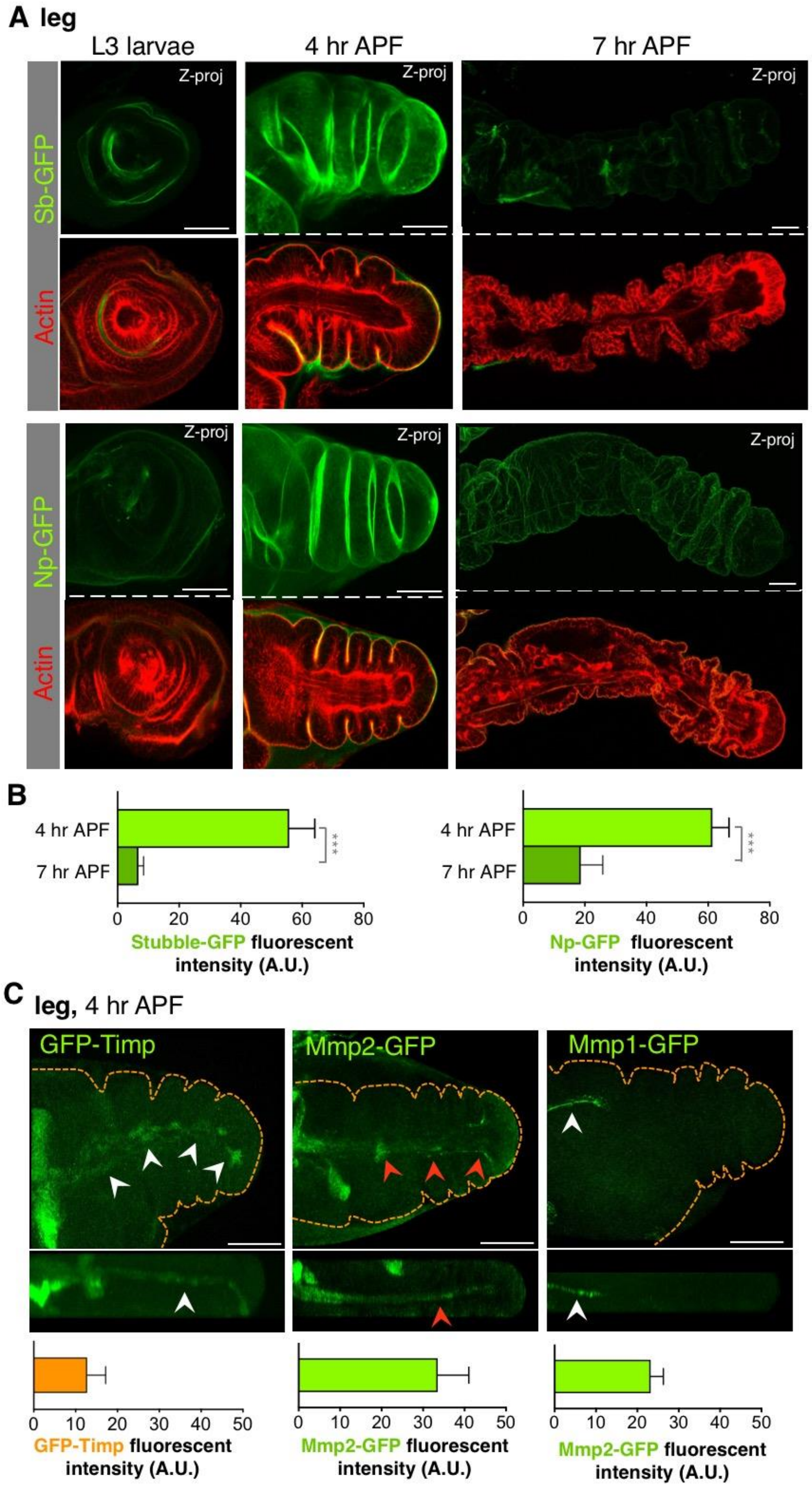

Figure S1. Sb, Np, Timp, MMP1 and MMP2 expression in the *Drosophila* pupal leg. (A) Sb-GFP and Np-GFP are still expressed in the legs at 4hr APF, consistent with the subsequent morphogenetic elongation of the leg. (B) Quantification of A. (C) GFP-Timp, Mmp2-GFP and Mmp1-GFP expression in the leg tendon/trachea.

Development • Supplementary information

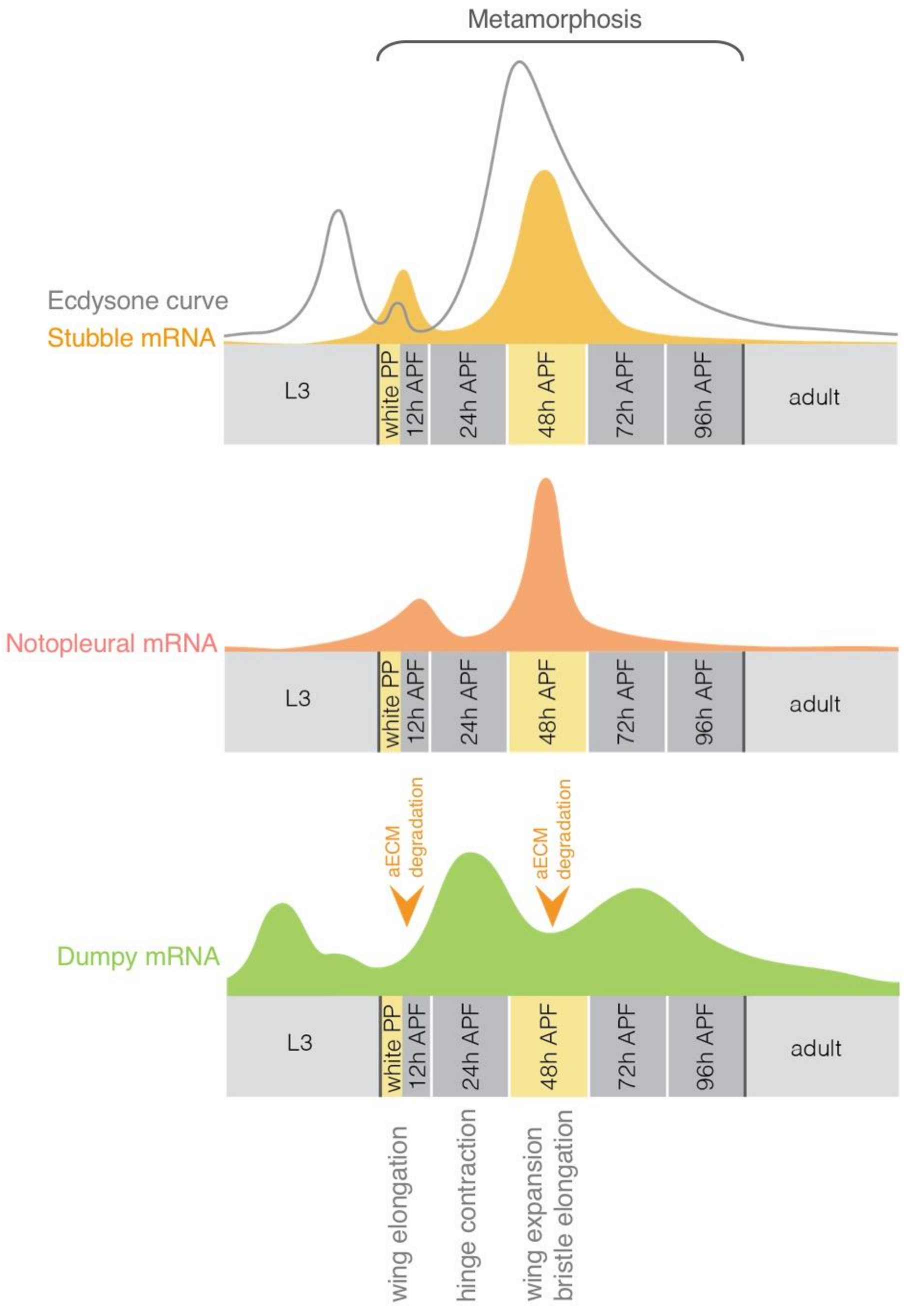

Figure S2. Stubble, Notopleural and Dumpy mRNA levels during metamorphosis. Expression profiles of mRNA for Sb, Np and Dp across pupal development were obtained from Flybase and suggest redundancy between Sb and Np, particularly during the second event of aECM remodelling at 48h APF.

Development • Supplementary information

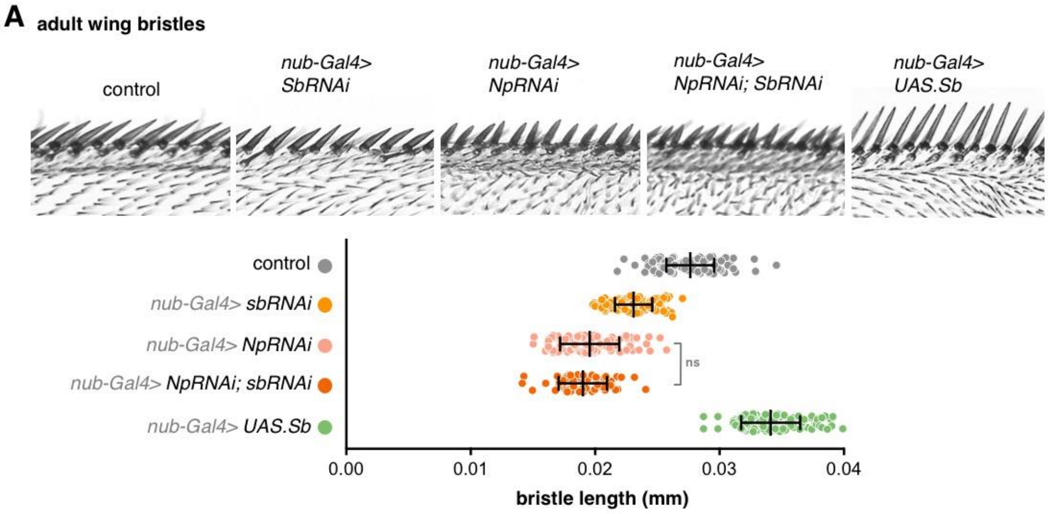

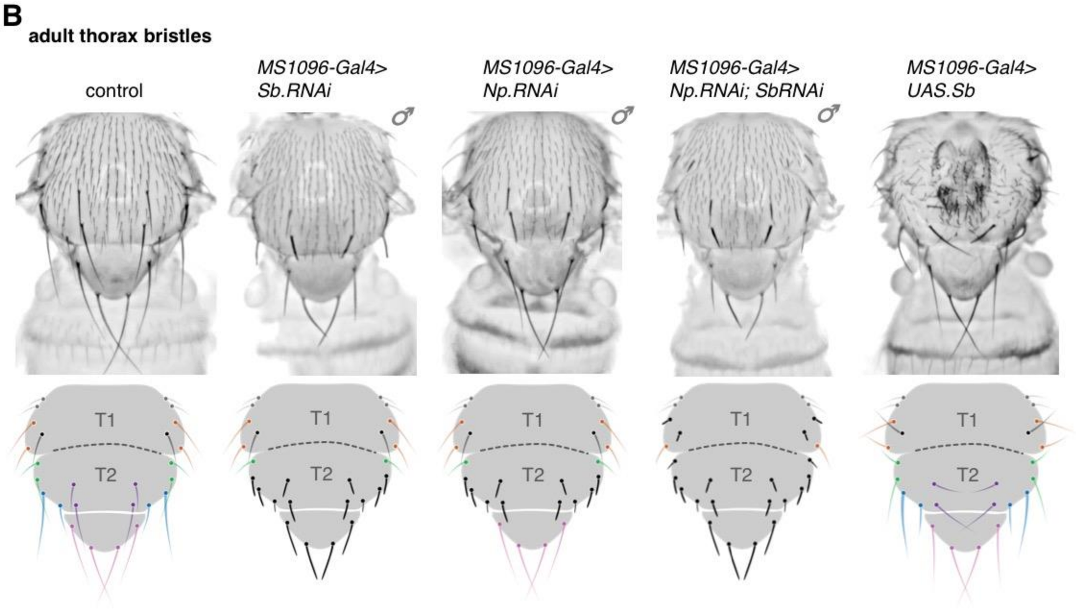

Figure S3. Stubble and Notopleural degrade Dumpy at late metamorphosis to elongate bristles at the wing margin and the thorax.

A) Adult Drosophila wing margin bristles shown at high magnification. Silencing of both Sb and Np by RNAi causes a shortened bristle phenotype, while overexpression of Sb increases bristle length.

B) Adult Drosophila thorax bristles are shown at low magnification and schematized below. Silencing of both Sb and Np by RNAi causes a shortened bristle phenotype, while overexpression of Sb increases bristle length and causes abnormal tissue morphogenesis and consequently abnormal bristle planar polarity

Development • Supplementary information

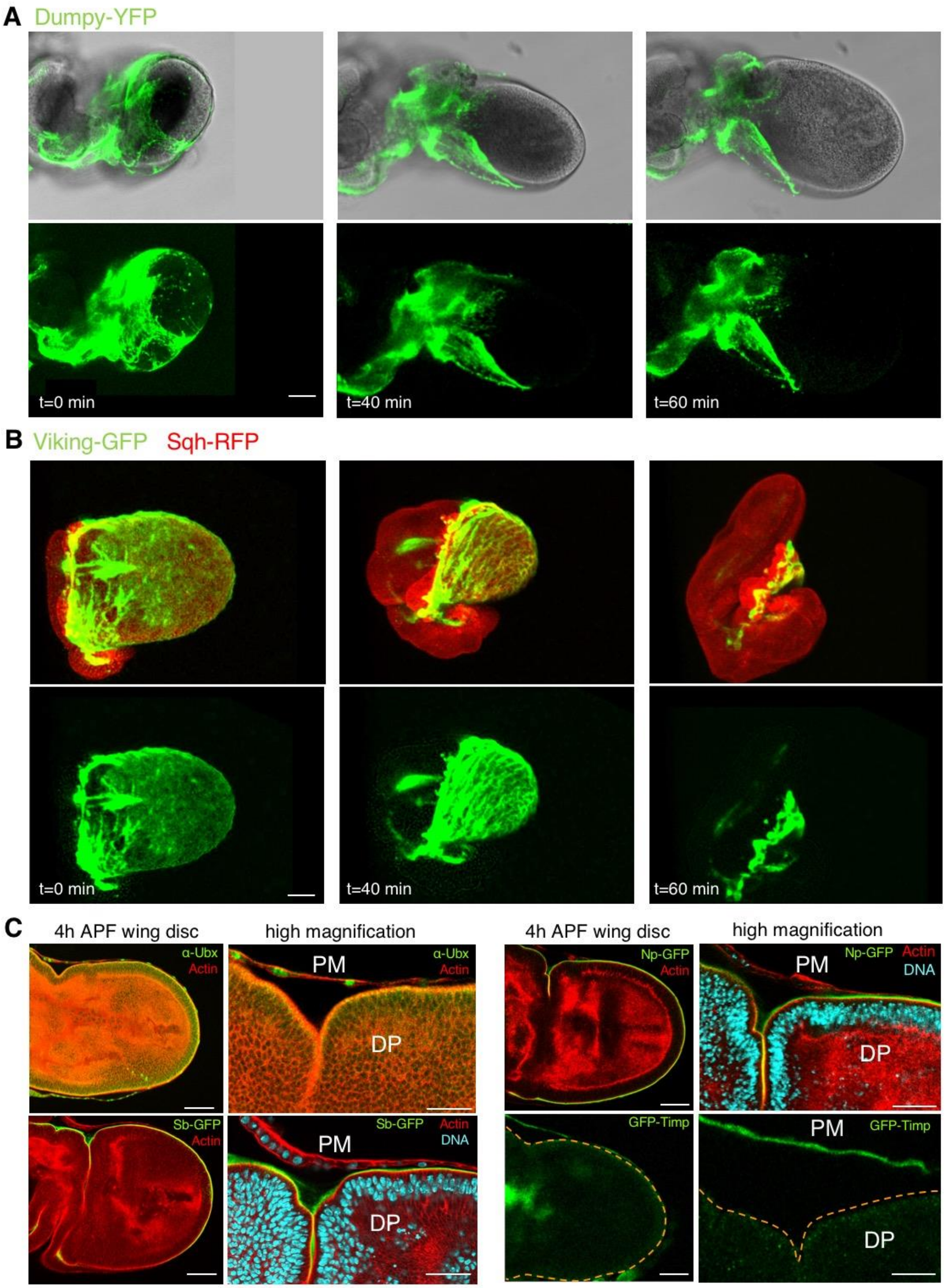

Figure S4. Maintenance of aECM and bECM during peripodial membrane retraction correlates with expression patterns of Ubx, Sb-GFP, Np-GFP and GFP-Timp.
A) Live imaging of Dp-YFP labelled aECM reveals maintenance of integrity during retraction.
B) Live imaging of Vkg-GFP labelled bECM reveals maintenance of integrity during retraction.
C) Fixed 4h APF wing discs showing repression of Sb-GFP and Np-GFP and expression of Ubx and GFP-Timp specifically in the peripodial membrane epithelium (PM).

Development • Supplementary information

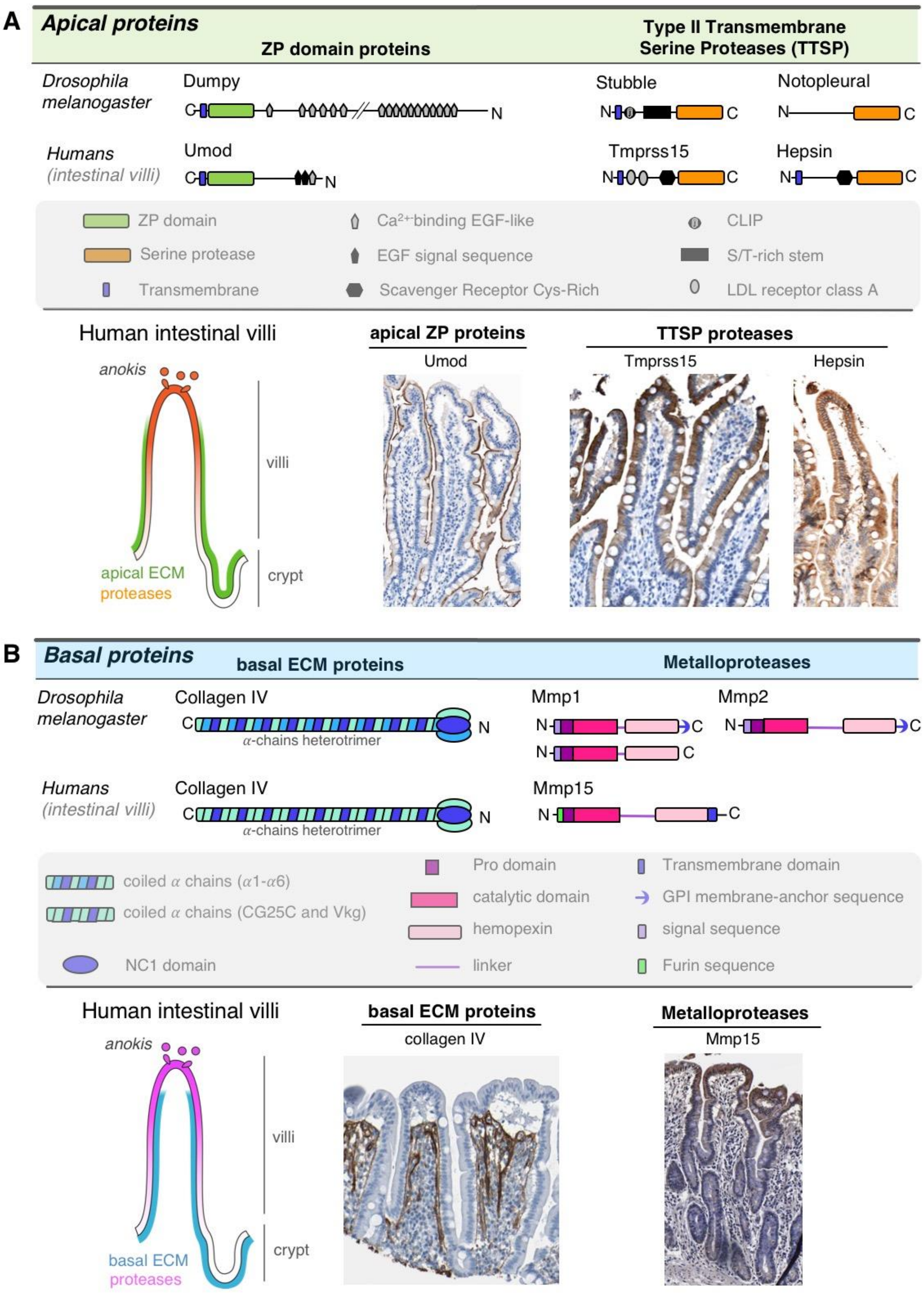

Figure S5. Basal and apical ECM and protease localisation along human intestinal villi.
A) Schematic diagram of aECM proteins and proteases and their expression patterns in villi.
B) Schematic diagram of bECM proteins and proteases and their expression patterns in villi.

Development • Supplementary information

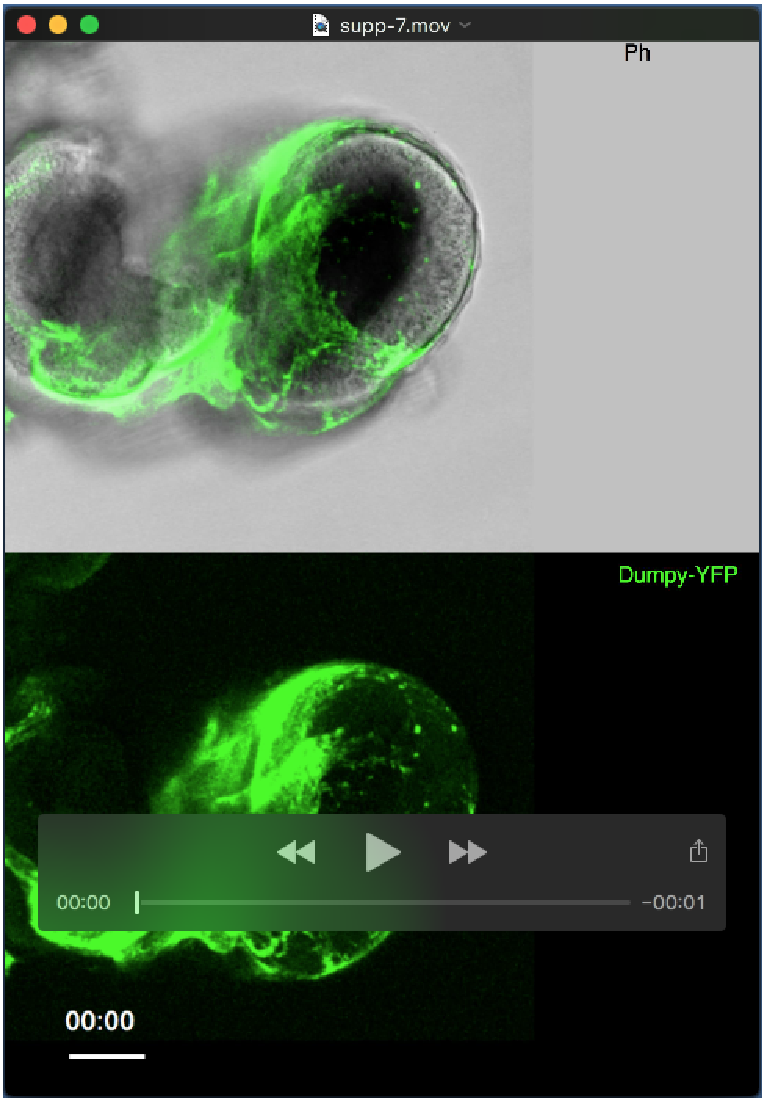

Movie 1. Degradation of Dumpy-YFP initiates wing disc eversion.

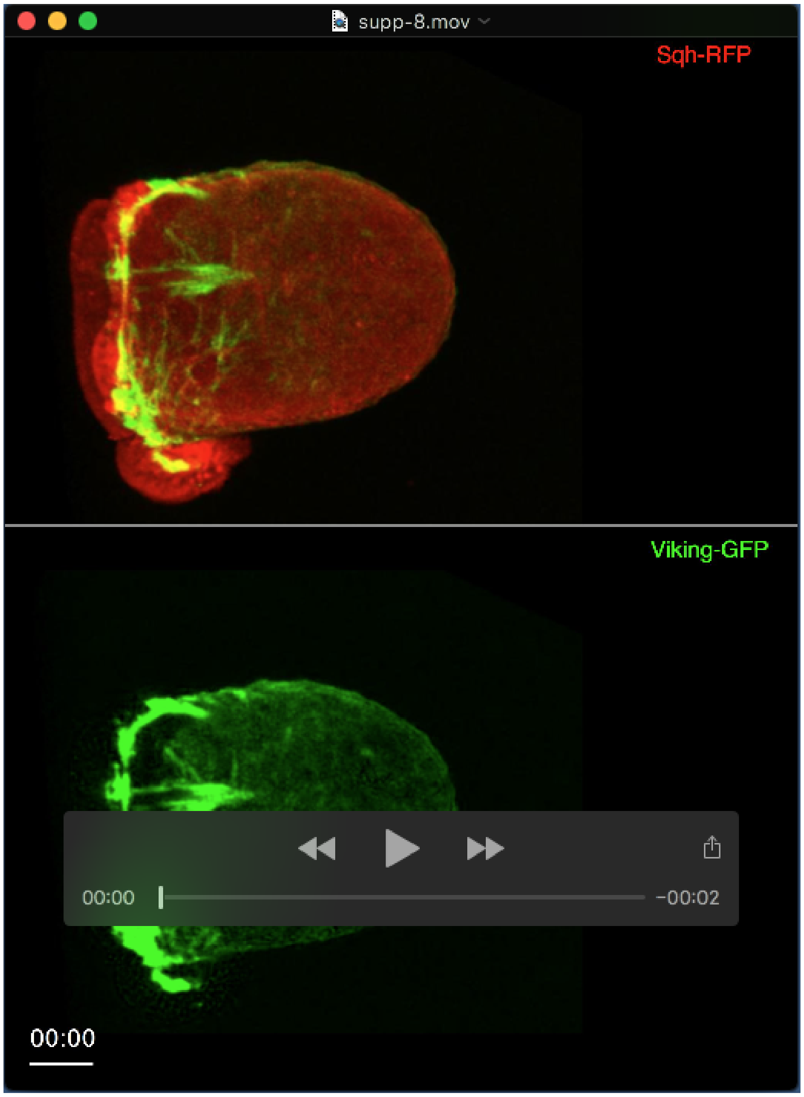

Movie 2. Degradation of Viking-GFP initiates wing disc eversion.
