# Supplementary material for: Control of tissue morphogenesis by the HOX gene Ultrabithorax
Source: Development. 2020 Mar 2;147(5):dev184564. doi: 10.1242/dev.184564 (PMC7063672; doi:10.1242/dev.184564)
Supplement: Reviewer comments [file develop-147-184564_review_history.pdf]

Control of tissue morphogenesis by the HOX gene *Ultrabithorax*

Maria-del-Carmen Diaz-de-la-Loza, Ryan Loker, Richard S. Mann and Barry J. Thompson
DOI: 10.1242/dev.184564

Editor: Thomas Lecuit

Review timeline
Original submission: 5 September 2019
Editorial decision: 1 November 2019
First revision received: 6 December 2019
Accepted: 28 January 2020

Original submission

First decision letter

MS ID#: DEVELOP/2019/184564

MS TITLE: The morphogenetic basis of homeotic transformation in *Drosophila*

AUTHORS: Maria-del-Carmen Diaz-de-la-Loza and Barry J. Thompson

I have now received all the referees reports on the above manuscript, and have reached a decision. The referees' comments are appended below, or you can access them online: please go to BenchPressand click on the 'Manuscripts with Decisions' queue in the Author Area.

The overall evaluation is clearly positive and we would like to publish a revised manuscript in Development, provided that the referees' comments can be satisfactorily addressed. In addressing the first point from Reviewer 1, I do not think necessary to invest too much in quantifications beyond simple metrics (eg. area, length and width). Please attend to the reviewers' comments in your revised manuscript and detail them in your point-by-point response. If you do not agree with any of their criticisms or suggestions explain clearly why this is so.

Reviewer 1

Advance summary and potential significance to field

The manuscript by Diaz-de-la-Loza and Thompson addresses the mechanism by which Ubx expression in the Drosophila haltere contributes to the characteristic haltere shape, as opposed to the wing shape. Previous work, including from the authors, have shown a requirement for apical ECM remodeling for proper wing elongation during pupal development. Now, the authors have analyzed, using CRIPR/Cas9 mediated knock-in lines, the expression of two aECM proteases, Sb and Np, in pupal wings and halteres. The authors show that Sb and Np are expressed during early pupal development in the wing, but to a lesser extent in the haltere. This partial repression is due to the expression of Ubx in the haltere. Data from previously published genome-wide Chip analyses are shown to indicate that Ubx directly binds within the Sb and Np genes. RNAi mediated knockdown of both genes result in small and roundish wings. Moreover, the authors have analyzed an inhibitor of metalloproteinases, Timp, that remodel basal ECM. Timp, again using a CRIPR/Cas9 mediated knock-in line, is expressed in the haltere, but not in the wing. RNAi mediated knockdown of Ubx results in reduced Timp expression. Overexpression of Timp in wings results in a slightly reduced wing size. In conjunction with knockdown of Sb and Np, Timp overexpression leads to small and

© 2020. Published by The Company of Biologists under the terms of the Creative Commons Attribution License (https://creativecommons.org/licenses/by/4.0/).

roundish wings. The authors propose a model in which expression of Ubx in halteres represses both aECM and bECM remodeling, and thereby prevents the transformation of the haltere into a wing.

The identification of a differential expression in halters and wings of Sb, Np, and Timp, and the control of these genes by Ubx, provides a significant advance in our understanding of homeotic haltere/wing transformations and may thus be of interest to a wider community. The presented data are by-and-large clear and convincing. Overall, I support publication of this manuscript. However, there are a number of points that I recommend the authors to address in order to strengthen their conclusions.

Comments for the author

Major concerns

First, to what extent does loss of ECM remodeling in wings leads to a haltere-like shape? The authors characterize the shape of wings as ‘elongated and flat’ and of halteres as ‘stumpy’ (line 75-76). The authors should characterize the shape differences between wild-type adult wings and halteres more in depth, preferably quantitatively, (e.g. elongation, flatness, etc) and then test whether wings in which ECM remodeling is compromised take on shape characteristics of a haltere.

Second, is repression of Sb and Np, or expression of Timp, required for normal haltere shape? The authors manipulate expression of these genes in the pupal wing, but not in the pupal haltere. The authors should express Sb and/or Np, or knockdown Timp, during haltere development.

Minor comments
- The title of the manuscript appears to be too general. The title may include the words Ubx, wing, haltere.

- Addition of subtitles to the Result part may enhance readability.

- Fig. 1A-C, Line 96 The authors claim that Sb-GFP and Np-GFP become expressed in the wing primordium at the end of larval instar. However, while there is some signal in the hinge folds, there is very little, if any, GFP signal in the larval wing disc pouch, the region of the wing disc that gives rise to the adult wing. Moreover, Fig. 1A-C show a strong decline of Sb-GFP and Np-GFP expression between 4 h and 7 h APF, but the authors do not mention this in the manuscript text. The authors should more carefully describe the data shown in Fig. 1A-C.

- Fig. 1BC, line 99 The authors state that Sb-GFP and Np-GFP are specifically repressed in the haltere. That seems to be an overstatement. ‘Partially repressed’ appears to be a more appropriate description of the data.

Line 111 ‘aECM remodeling” might not be the appropriate term; what is shown is that silencing of Sb leads to higher levels of Dp-GFP/aECM.

Fig. 3A-D line 121 The authors should describe the experimental data shown in this figure.

Fig. 3 The authors should mention what is shown in the red channel.

Fig. 4 The authors should describe in more detail the experiments leading to the data shown in Fig. 4.

Line 131 The authors should mention that Collagen IV is encoded by vkg and Col4A1/Cg25C, and not only by vkg.

Line 141/2 Silencing of Ubx causes a corresponding loss…. Should refer to Fig. 5C, not Fig. 5B.

Fig. 5C The authors should show a control for 7hr APF.

© 2020. Published by The Company of Biologists under the terms of the Creative Commons Attribution License (https://creativecommons.org/licenses/by/4.0/).

Line 155 The authors claim that '…producing a small stump approximating the appearance of Ubx overexpressing wings'. The authors should clarify what they mean by appearance. The Ubx overexpressing wing is clearly much smaller.

Fig. 7 The authors propose that the control of Ubx on Sb, Np and Timp expression contributes to the shape difference between wings and halters. The silencing of Sp and Np combined with Timp overexpression produces a small, roundish wing, indicating that aECM and bECM remodeling is not only involved in the shaping of the wing, but also in the growth of the wing. The authors should discuss this issue.

Reviewer 2

Advance summary and potential significance to field

Diaz-de-la-Loza and Thompson present a beautiful result. Few phenotypes in biology are more enigmatic than the four winged fly resulting from a homeotic transformation of a haltere to a wing due to lack of Ubx expression. The process has attracted substantial attention, including the Nobel committee. Yet, even after decades of studies of the target genes of the Ubx homeobox transcription factor, we do not understand how Ubx represses the morphogenesis of the beautiful fly wing to produce the much less spectacular haltere. This reflects a broader problem in contemporary developmental biology, that is, the strong disconnect between the understanding of patterning mechanisms and the effector genes and physical processes that shape tissues during morphogenesis. The authors of the presented study establish one such connection in a convincing manner. They show that amongst the Ubx target genes, perhaps surprisingly, the proteases affecting the remodelling of apical and basal extracellular matrix (eECM) are at least in part responsible for the wing like morphogenesis. Ubx represses two of the proteases in apical ECM and concurrently upregulates the inhibitor of another family of proteases remodelling basal ECM. When the two apical proteases are downregulated in the segment bearing wing, the wing expansion is dramatically stunted. Loss of Ubx in the haltere leads to a loss of expression of matrix metalloprotease inhibitor Timp. When the two conditions are combined, i.e. apical ECM proteases RNAi with over-expression of the inhibitor Timp in the wing-bearing segment, the reduction of the wing is even stronger. It starts to resemble the phenotype induced by over-expression of Ubx itself. This suggests that the authors are certainly on the right track towards understanding how mechanistically Ubx mediated homeotic transformation works. The authors have generated number of useful reagents to visualize the expression of the various players in this story. They even show that analogous patterned ECM remodelling occurs in the intestinal crypts and villi of mammals. The finding has major evolutionary implications and points towards a previously neglected morphogenetic mechanism operating through ECM remodelling. Linking it to Ubx regulation is the cherry on the cake.

Comments for the author

I reviewed this paper before at another journal and my comments have been addressed. I have nothing to add.

First revision

Author response to reviewers' comments

Dear Thomas,

We hereby re-submit our revised manuscript, now entitled "Control of tissue morphogenesis by the HOX gene Ultrabithorax" to Development.

© 2020. Published by The Company of Biologists under the terms of the Creative Commons Attribution License (https://creativecommons.org/licenses/by/4.0/).

We are grateful to the reviewers for their strong interest in the manuscript and for their constructive comments. Reviewer 1 states that “The identification of a differential expression in halteres and wings of Sb, Np, and Timp, and the control of these genes by Ubx, provides a significant advance in our understanding of homeotic haltere/wing transformations and may thus be of interest to a wider community. The presented data are by-and-large clear and convincing. Overall, I support publication of this manuscript.” and Reviewer 2 states that “The finding has major evolutionary implications and points towards a previously neglected morphogenetic mechanism operating through ECM remodelling” and “I reviewed this paper before at another journal and my comments have been addressed”. However, Reviewer 1 felt that “there are a number of points that I recommend the authors to address in order to strengthen their conclusions”. In our revised manuscript, we provide substantial new data to address all of the comments Reviewer 1.

In addition to the reviewer’s comments, we have improved our work by expanding the data on Ubx binding to the genes involved in ECM degradation. In collaboration with Ryan Loker in Richard Mann’s laboratory, we have included new chromatin immunoprecipitation data showing Ubx binding sites to chromatin extracted from third instar larvae halteres. The new data have been organised in two figures, Figure 4 (apical Sb and Np) and Figure 6 (basal Timp, Mmp1 and Mmp2), and show new Ubx binding sites to Sb and Np (in addition to the previous published ones in [1, 2], and for the first time Ubx binding sites to the Timp, Mmp1 and Mmp2 genes involved in basal ECM degradation.

Reviewer 1 Major Concerns:
To what extent does loss of ECM remodelling in wings leads to a haltere-like shape? The authors characterize the shape of wings as ‘elongated and flat’ and of halteres as ‘stumpy’ (line 75-76). The authors should characterize the shape differences between wild-type adult wings and halteres more in depth, preferably quantitatively, (e.g. elongation, flatness, etc) and then test whether wings in which ECM remodeling is compromised take on shape characteristics of a haltere.
As requested, we have now quantified the characteristics that differentiate wings from halteres, area and shape, in all genotypes analysed comparing them with control halteres. We have included the new data in the Figure 2 (Fig. 2C) and Figure 7 (Fig. 7C) along with the appropriate genotypes, and described them in the text. For the wing blade or the haltere we have calculated their total area. To characterise shape, we have analysed three aspects: elongation by calculating the aspect ratio (maximum length/maximum width), dorsoventral adhesion (percentage of inflated area), and the number of samples which have folded epithelia. In Figure 7C, additionally to control halteres, we have included the analysis of wing expressing the UbxIa allele (wing to haltere partial transformation), and halteres expressing Ubx.RNAi (haltere to wing partial transformation).
The new data allowed us to better describe the phenotypic transformation from wings to halteres caused by the impairment of ECM degradation. Basal and apical protease depletion and/or Timp overexpression change wings to acquire haltere-like characteristics: smaller area, rounder shape, and dorsal and ventral layers not completely adhered. Wing phenotypes increase as more components of the ECM degradation inhibition are depleted. The area of mutant wings decreases up to 3 times compared to control wings, depletion of Sb and Np is sufficient to generate rounder wings with the same aspect ratio than halteres, and in most mutant conditions half or more of the wing blade surface is inflated. As expected, the area of ECM degradation depleted wings is always bigger than control halteres, since in the haltere Ubx also reduces cell proliferation. Finally, all mutant combinations lead to wing folding, which contributes to defects of wing elongation and the decrease of wing area.
Is repression of Sb and Np, or expression of Timp, required for normal haltere shape? The authors manipulate expression of these genes in the pupal wing, but not in the pupal haltere. The authors should express Sb and/or Np, or knockdown Timp, during haltere development.
We were indeed very interested in the effect of ectopic degradation of ECM in the haltere, and we designed and perform a set of experiments to express both UAS.Mmp2 and UAS.Sb and to examine haltere morphogenesis in that situation. However, we found those conditions were always lethal before the third instar larval stage, likely due to ectopic ECM degradation in the embryo (Ubx.Gal4 is expressed in the T3 segment from early in embryogenesis). Lethality could not be rescued by use of a Ubx-Gal4, tub-gal80 driver line to reduce Gal4 activity during embryogenesis, preventing us from analysing the resulting adult halteres. Nevertheless, our previous manuscript (Dev Cell 2018) showed that degradation of the ECM in larval halteres by limited treatment with the proteolytic enzyme trypsin in culture was sufficient to induce expansion and flattening of the haltere disc into a more wing-like morphology.

© 2020. Published by The Company of Biologists under the terms of the Creative Commons Attribution License (https://creativecommons.org/licenses/by/4.0/).

Reviewer 1 Minor Concerns:
The title of the manuscript appears to be too general. The title may include the words Ubx, wing, haltere.
We agree with this point, and have changed the title to “Control of tissue morphogenesis by the HOX gene Ultrabithorax” to make it more specific.

Addition of subtitles to the Result part may enhance readability.
Done.

Fig. 1A-C, Line 96 The authors claim that Sb-GFP and Np-GFP become expressed in the wing primordium at the end of larval instar. However, while there is some signal in the hinge folds, there is very little, if any, GFP signal in the larval wing disc pouch, the region of the wing disc that gives rise to the adult wing. Moreover, Fig. 1A-C show a strong decline of Sb-GFP and Np-GFP expression between 4 h and 7 h APF, but the authors do not mention this in the manuscript text. The authors should more carefully describe the data shown in Fig. 1A-C.
To better describe Sb and Np expression in the wing in Fig 1A-C, we have explained in detail the pattern of expression of apical proteases Sb and Np, including annotations in Fig1A and Fig 1A legend to differentiate the hinge and the pouch areas in 3rd instar larvae wing discs.

Fig. 1BC, line 99 The authors state that Sb-GFP and Np-GFP are specifically repressed in the haltere. That seems to be an overstatement. ‘Partially repressed’ appears to be a more appropriate description of the data.
We have clarified in the text that Sb-GFP and Np-GFP are partially repressed in the haltere.

Line 111 ‘aECM remodeling” might not be the appropriate term; what is shown is that silencing of Sb leads to higher levels of Dp-GFP/aECM.
We have indicated in the text that Sb depletion results in higher levels of apical ECM in 7hr APF wings.

Fig. 3A-D line 121 The authors should describe the experimental data shown in this figure.
We have described in the main text the experimental data shown in Figure 3A-D about the late event of apical ECM degradation to allow wing expansion.

Fig 3. The authors should mention what is shown in the red channel.
We have indicated that we are visualising the actin cytoskeleton in red in Figure 3 and in Figure 3 legend.

Fig 4. The authors should describe in more detail the experiments leading to the data shown in Fig 4.
We have described Figure 4 in more detail in the main text and in Figure legend 4. Also, we have included new chromatin immunoprecipitation data from Ryan Loker in Richard Mann laboratory, extending Figure 4 and creating a new Figure 6 for Timp, Mmp1 and Mmp2.

Line 131. The authors should mention that Collagen IV is encoded by vkg and Col4A1/Cg25C, and not only by vkg.
Done.

Line 141/2. Silencing of Ubx causes a corresponding loss…. Should refer to Fig. 5C, not Fig. 5B.
Corrected.

Fig 5C. The authors should show a control for 7hr APF.
We have included 4hr and 7hr APF control halteres images in Figure 5C.

Line 155 The authors claim that ‘…producing a small stump approximating the appearance of Ubx overexpressing wings’. The authors should clarify what they mean by appearance. The Ubx overexpressing wing is clearly much smaller.
We have extended the text describing the results shown in Figure 7 to be more precise about the adult wing phenotypes in nubG4>NpRNAi; SbRNAi, UASTimp flies when compared with UbxIa

© 2020. Published by The Company of Biologists under the terms of the Creative Commons Attribution License (https://creativecommons.org/licenses/by/4.0/).

expressing wings, including references to the new area and shape quantification data shown in new Figure 7C.

Fig. 7 The authors propose that the control of Ubx on Sb, Np and Timp expression contributes to the shape difference between wings and halteres. The silencing of Sp and Np combined with Timp overexpression produces a small, roundish wing, indicating that aECM and bECM remodeling is not only involved in the shaping of the wing, but also in the growth of the wing. The authors should discuss this issue.
We have discussed in the text the differences in area when comparing mutant wings overexpressing Sb, Np and Timp with Ubxla expressing wings and control halteres using the characterisation of adult phenotypes in the wing and the haltere shown in new Figure 7. Depletion of ECM degradation most likely reduces wing size by affecting wing expansion and elongation in early and late metamorphosis, which also fold the wings, and by impairing the adhesion of dorsal and ventral wing layers, leading to the presence of blisters. We don't have evidence for reduced cell numbers or size in these wings, so wish to be careful about referring to tissue growth in our conclusions.

References

1. Slattery, M., Ma, L., Negre, N., White, K.P., and Mann, R.S. (2011). Genome-wide tissue-specific occupancy of the Hox protein Ultrabithorax and Hox cofactor Homothorax in Drosophila. PLoS One 6, e14686.
2. Choo, S.W., White, R., and Russell, S. (2011). Genome-wide analysis of the binding of the Hox protein Ultrabithorax and the Hox cofactor Homothorax in Drosophila. PLoS One 6, e14778.

Second decision letter

MS ID#: DEVELOP/2019/184564

MS TITLE: Control of tissue morphogenesis by the HOX gene *Ultrabithorax*

AUTHORS: Maria-del-Carmen Diaz-de-la-Loza, Ryan Loker, Richard S. Mann, and Barry J. Thompson
ARTICLE TYPE: Research Article

I am happy to tell you that your manuscript has been accepted for publication in Development, pending our standard ethics checks.

Reviewer 1

Advance summary and potential significance to field

The paper provides an important advance in our understanding of how the ubx gene controls morphogenesis.

Comments for the author

The authors have satisfactorily addressed my comments. There are a few minor issues the authors may wish to clarify.

1. Fig. 2C and 7C, "Size": The y-axis is labelled "wing blade/haltere area". I believe the authors have either measured the area of the wing blade or the area of halteres. The way they have written it, however, may be interpreted as a ratio of wing blade area to haltere area. "Wing blade or haltere area" might be more appropriate.
2. Fig. 2C and 7C, "Shape": The label on the y-axis "folded wings" may not be appropriate, in particular because the authors have measured this parameter in halteres.

© 2020. Published by The Company of Biologists under the terms of the Creative Commons Attribution License (https://creativecommons.org/licenses/by/4.0/).

3. Fig. 2C and 7C. The legends should indicate what is shown (mean and standard deviation?) and also the number of wings and halteres analyzed.

Reviewer 2

Advance summary and potential significance to field

This paper is ready for primetime.

Comments for the author

n.a.

© 2020. Published by The Company of Biologists under the terms of the Creative Commons Attribution License (https://creativecommons.org/licenses/by/4.0/).
